# Advancing Maxillary Reconstruction: A Systematic Review and Meta-Analysis of the Evolving Role of the Scapular Free Flap

**DOI:** 10.3390/jcm14103278

**Published:** 2025-05-08

**Authors:** Giovanni Salzano, Veronica Scocca, Stefania Troise, Vincenzo Abbate, Paola Bonavolontà, Luigi Angelo Vaira, Alfonso Scarpa, Jerome R. Lechien, Gianluca De Fazio, Emanuele Carraturo, Giovanni Dell’Aversana Orabona

**Affiliations:** 1 Maxillofacial Surgery Unit, Department of Neurosciences, Reproductive and Odontostomatological Sciences, University Federico II, Via Pansini 5, 80131 Naples, Italy; giovannisalzanomd@gmail.com (G.S.); stefania.troise@unina.it (S.T.); vincenzo.abbate@unina.it (V.A.); gianludefazio@gmail.com (G.D.F.); emanuele.c2971995@gmail.com (E.C.); giovanni.dellaversanaorabona@unina.it (G.D.O.); 2 Maxillofacial Surgery Unit, Department of Clinical and Surgical Medicine, University Federico II, Via Pansini 5, 80131 Naples, Italy; paola.bonavolonta@unina.it; 3 Maxillofacial Surgery Operative Unit, Department of Medical, Surgical and Experimental Sciences, University of Sassari, 07100 Sassari, Italy; luigi.vaira@gmail.com; 4 Department of Medicine, Surgery and Dentistry, University of Salerno, 84081 Salerno, Italy; ascarpa@unisa.it; 5 Department of Otolaryngology and Head and Neck Surgery, Division of Laryngology and Broncho-Esophagology, UMONS Research Institute for Health Sciences and Technology, EpiCURA Hospital, University of Mons, 7301 Mons, Belgium; lechienj@gmail.com

**Keywords:** scapular free flap, maxillectomy, midface, morbidity, functional outcomes, systematic review, meta-analysis

## Abstract

**Background/Objectives:** This systematic review and meta-analysis evaluates the surgical, functional, and aesthetic outcomes of scapular free flaps in maxillary reconstruction. The primary objective is to assess early surgical complications, fistula formation, donor site morbidity, dental restoration, normal dietary intake, aesthetic compromise, and eye-related issues. Secondary objectives include total free flap necrosis, the need for revision procedures, and functional performance of the upper limb. **Methods:** A systematic review was conducted following the PRISMA guidelines. Eligible studies were identified by searching PubMed/MEDLINE, Cochrane Library, Scopus, and Google Scholar, with the last search conducted on 10th February 2025. Inclusion criteria were studies reporting on patients undergoing maxillary reconstruction with scapular free flaps, and which provided data on at least one of the primary or secondary outcomes. A single-arm meta-analysis was performed to assess the outcomes of scapular free flap reconstruction. The risk of bias was assessed using the Newcastle–Ottawa Quality Assessment Scale, with two independent reviewers performing the assessment. **Results:** From an initial search of 310 articles, 6 studies were included in the qualitative and quantitative synthesis, encompassing 231 patients with a mean age of 52.9 years (95% CI 44.9–60.8). Early general surgical complications occurred in 24% (95% CI 13–40) of patients, while 12% (95% CI 4–31) experienced fistula formation. Donor site morbidity was reported in 10% (95% CI 6–17) of cases, with a mean DASH score of 10.49, indicating low upper limb impairment. Dental rehabilitation was achieved in 56% (95% CI 42–70), and 52% (95% CI 31–72) of patients resumed a normal diet. Aesthetic compromise was observed in 27% (95% CI 9–58), and 36% (95% CI 28–44) reported eye-related issues. **Conclusions:** Scapular free flap is a reliable option for maxillary reconstruction with favourable outcomes, particularly in complex composite defects requiring both bone and soft tissue reconstruction. However, the evidence is limited by risk of bias, significant heterogeneity, and imprecision due to the small number of studies and participants. Larger, more robust trials are needed to confirm these findings.

## 1. Introduction

Midface reconstruction remains a significant challenge in head and neck surgery. Traumas and the treatment of tumors affecting the maxilla result in defects involving not only the bone but also the surrounding soft tissues, orbital contents, and nasal structures. These defects can have a profound impact on both function and aesthetics. Additionally, maxillary reconstruction is complicated by factors such as prior scars, skin involvement, and radiation therapy. Thus, the restoration of the midfacial anatomy, facial symmetry, and function requires careful planning and the selection of appropriate reconstructive techniques [1,2,3].

The primary objectives of maxillary reconstruction include restoring the three-dimensional structure of the midface, maintaining facial symmetry, supporting the orbital contents, separating the oral and nasal cavities, and providing a stable base for dental rehabilitation [4,5,6].

Multiple reconstructive approaches have been explored, with fibula and iliac crest (deep circumflex iliac artery) free flaps being among those most commonly employed. These techniques have demonstrated their effectiveness in restoring bony continuity and soft tissue coverage [6,7,8,9]. However, they come with limitations, including donor site morbidity, the potential for insufficient soft tissue coverage, and challenges in achieving optimal cosmetic outcomes [10,11].

In recent years, the scapular free flap (SFF) has emerged as a viable alternative due to its favorable characteristics. The scapular tip, with its resemblance to the native maxilla, provides an ideal option for reconstructing palate alveolar defects, often in combination with zygomaticomaxillary buttress and orbital floor reconstruction. Additionally, the SFF offers minimal donor site morbidity, a consistent vascular anatomy, an extended pedicle, and the potential for chimeric flap design. Chimeric flaps allow for the inclusion of different tissue components, such as bone, muscle, and skin, making them particularly useful for addressing complex defects involving multiple tissue types. This flexibility is a significant advantage, especially in cases where an extensive reconstruction of both bony and soft tissue structures is required [12,13,14,15,16].

This systematic review and meta-analysis assesses the functional and aesthetic outcomes of the SFF in maxillary reconstruction, focusing on donor site morbidity, dental rehabilitation, dietary intakes, aesthetic and eye problems, and upper limb function. This study aims to contribute to a clearer understanding of the role of the SFF in maxillary reconstruction and to support clinical decision-making by summarizing the available evidence on surgical, functional, and aesthetic outcomes.

## 2. Materials and Methods

This study followed the Preferred Reporting Items for Systematic Reviews and Meta-Analyses (PRISMA) guidelines [17]. Since it involved a review of previously published studies, neither ethics approval nor informed consent was required. Additionally, the review was registered in the PROSPERO database under the ID number **CRD420251039019**.

### 2.1. Search Strategy

The study search included PubMed/MEDLINE, Cochrane Library, Scopus, and Google Scholar. The search was conducted independently by two investigators (V.S. and G.S.). Relevant keywords, phrases, and MeSH terms were tailored to meet the specific requirements of each individual database (Table 1). An example of the search strategy is that used for PubMed/MEDLINE: “scapular flap” or “scapular free flap” and “maxillary reconstruction”. Next, a cross-reference search of the selected articles was conducted using the snowballing method to ensure the retrieval of all possible studies. One author (V.S.) independently compiled a standardized form to extract the following characteristics from the included studies: authors, year of publication, country, study design, number of patients, pathology, type of classification of the maxillary defects, use of neoadjuvant and/or adjuvant therapies, timing of surgery, type of flap used for reconstruction, flap orientation, mean follow-up time, objective outcomes, and Patient Reported Outcome Measures (PROMs). The accuracy of the extracted data was verified by a second author (G.S.).

The electronic database search was conducted from 2nd February 2025 to 10th February 2025.

### 2.2. Eligibility Criteria

This systematic review and meta-analysis was carried out in accordance with the PICOTS: Patients (P), patients who had undergone SFF reconstruction of a post-ablative maxillectomy defect for any reason (e.g., cancer, trauma); Intervention (I), total or partial maxillectomy with SFF reconstruction; Comparison (C), none; Outcomes (O) surgical, functional and aesthetic outcomes; Timing (T), none; Study design (S), retrospective and prospective cohort studies, case–control and cross-sectional studies, and randomized clinical trials (RCTs). The inclusion and exclusion criteria are presented in Table 2. No age limits were applied to the studies’ population, and no restrictions on the publication date were imposed, ensuring the inclusion of relevant studies from any time period.

### 2.3. Data Collection Process

References from the identified databases were merged, and duplicates were removed using the reference management software EndNote^®^ 21 (version 21.5). The articles were screened for relevance based on title and abstract, with those deemed appropriately selected for full-text review. Any discrepancies between the screening authors were resolved through discussion until a consensus was achieved.

Systematic data extraction from the included studies was made using a structured form, with data archived in a customized Excel^®^ (Microsoft Corp, Seattle, WA, USA) spreadsheet.

#### Outcomes Measures

Regarding the surgical outcomes investigated:

- Early General Surgical Complications.
- Fistula Formation.

Regarding the functional outcomes analyzed:

- Donor Site Morbidity.
- Upper Limb Function using the DASH score.
- Dental Restoration.
- Diet.
- Aesthetic Compromise.
- Eye problems.

The DASH (Disabilities of the Arm, Shoulder and Hand) score is a self-reported questionnaire used to assess upper limb function. It measures the severity of physical symptoms and disabilities in the arm, shoulder, and hand. The questionnaire consists of 30 items that cover various activities of daily living, such as lifting objects, reaching, and carrying. The score is calculated on a scale from 0 to 100, with 0 indicating no disability and 100 indicating severe disability [18].

### 2.4. Risk of Bias and Study Quality Assessment

Two authors (V.S. and G.S.) assessed the quality of each study using the Newcastle–Ottawa Quality Assessment Scale [19], since all included studies were observational cohort or case–control studies. A sensitivity analysis was not conducted in this review due to the limited number of studies included in the meta-analysis. To evaluate any potential publication bias, a funnel plot was generated based on the effect size of each outcome.

### 2.5. Data Synthesis and Analysis

All statistical analyses were conducted using R software (version 4.4.2) with the “meta” and “dmetar” packages. Clinical outcomes were reported as provided by the individual studies. Categorical variables were summarized using counts and percentages, while continuous variables were presented as medians with 95% confidence intervals (CIs).

A single-arm meta-analysis was performed to evaluate early surgical complications, fistula formation, donor site morbidity, dental rehabilitation, normal dietary intake, aesthetic compromise, and eye-related problems. The results were reported as pooled estimates with 95% CIs, and forest plots were generated for each outcome. To stabilize variance in the analysis of proportions, the Freeman–Tukey double arcsine transformation was applied.

Cochran’s Q test was used to assess heterogeneity between studies, and I^2^ was calculated to measure the variation between studies. The I^2^ statistic represents the percentage of total variation between studies that is due to heterogeneity rather than chance. Based on the Cochrane criteria, I^2^ values were interpreted as follows: values from 0% to 40% indicate low heterogeneity, 30% to 60% represent moderate heterogeneity, 50% to 90% indicate substantial heterogeneity, and 75% to 100% suggest considerable heterogeneity. The 95% confidence interval for I^2^ was calculated using the Q statistic and its degrees of freedom.

A random-effects model was used for all meta-analyses, assuming that the true effect size may vary across studies due to differences in study populations, methodologies, or other sources of variability. This model accounts for both within-study and between-study heterogeneity, providing more conservative and generalizable effect estimates.

Publication bias was assessed by visually inspecting the funnel plot, and Egger’s linear regression test was used to statistically examine any asymmetry in the funnel plot, which could indicate publication bias. Statistical significance was defined as *p* < 0.05 for all tests. Given the limited number of studies, this assessment was considered exploratory in nature.

## 3. Results

### 3.1. Study Selection

**Figure 1:**
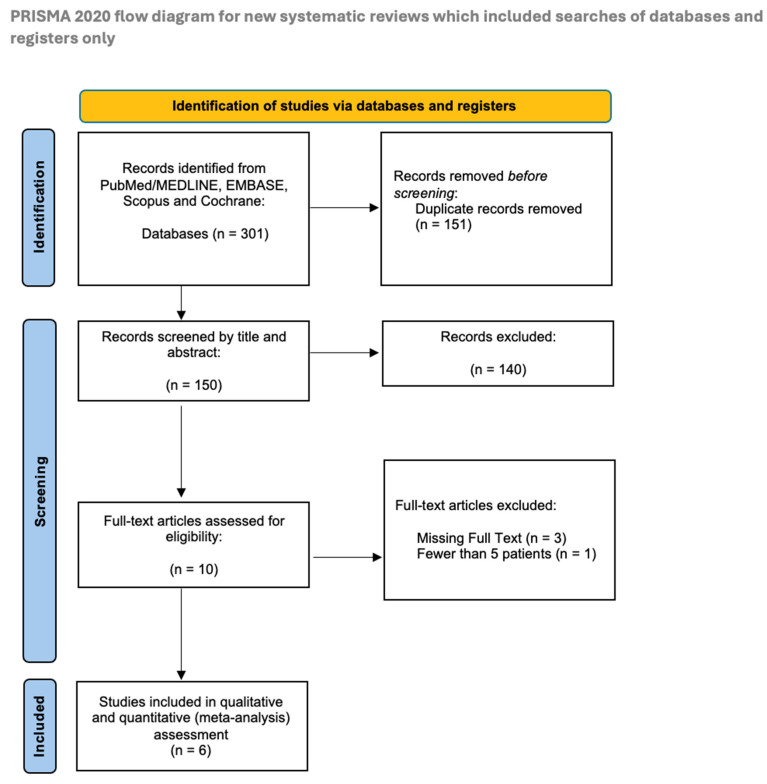
PRISMA flow diagram of study selection.

The study selection process is summarized in Figure 1. The data collection resulted in 905 entries. Out of 301 articles, 151 were excluded before screening because they were duplicates. After the initial screening of the titles and abstracts, 140 articles were excluded for reasons such as the use of non-scapular flaps, lack of relevant outcome data, insufficient data granularity, or being off-topic. The remaining 10 were included for full-text assessment. One article was excluded because it involved fewer than five patients [20], and three articles were excluded because of the missing full text [21,22,23]. Therefore, a total of six publications were included in the qualitative and quantitative (meta-analysis) synthesis [12,13,15,24,25,26].

### 3.2. Description of the Studies

The general characteristics of the studies are shown in Table 3. All the studies were in English, and all were retrospective except one that was prospective [21]. The countries in which the studies were conducted were Canada (*n* = 2) [12,13], Italy (*n* = 1) [24], France (*n* = 1) [15], Portugal (*n* = 1) [25], and Sweden (*n* = 1) [26]. One study was published in the 2000s [12], two in the 2010s [13,15], and three in the 2020s [24,25,26].

### 3.3. Study Results

A total number of 231 patients (males: 106/154, 68.8%) with a mean age of 52.9 years (*n* = 192/231, 95% CI 44.9–60.8) were included. Most of the patients presented a malignant condition (*n* = 208/231, 90%), especially squamous cell carcinoma. A total of 80 patients had undergone previous treatment before surgery (i.e., surgery, radiotherapy, and/or chemotherapy) (*n* = 80/178, 44.9%), and 145 patients received adjuvant radiotherapy (*n* = 145/231, 62.3%). In three studies, the maxillectomy defect was classified according to Brown’s classification [1,24,25,26], whereas two studies [12,13] used the Okay et al. classification [27] and one used the Kolb classification of facial defects [15]. According to the Brown classification [1], there were 17 class I cases (*n* = 17/94, 18.1%), 43 class II cases (*n* = 43/94, 45.7%), 33 class III cases (*n* = 33/94, 35.1%), and 1 class IV case (*n* = 1/94, 1.1%).

Three studies reported the flap orientation used for the reconstruction [12,24,25]. A horizontal orientation was used in 47 cases (*n* = 47/88, 53.4%), with a vertical orientation used in 41 cases (*n* = 41/88, 46.6%). Additionally, these studies reported the surgical timing. A total of 61 patients underwent primary reconstruction (*n* = 61/74, 82.4%) and 13 underwent delayed (secondary) reconstruction (*n* = 13/74, 17.6%). The mean length of hospitalization, reported only by three studies, was 17 days (95% CI 3.4–30.8) [12,15,25].

### 3.4. Meta-Analyses of the Surgical Outcomes and Complications

**Figure 2:**
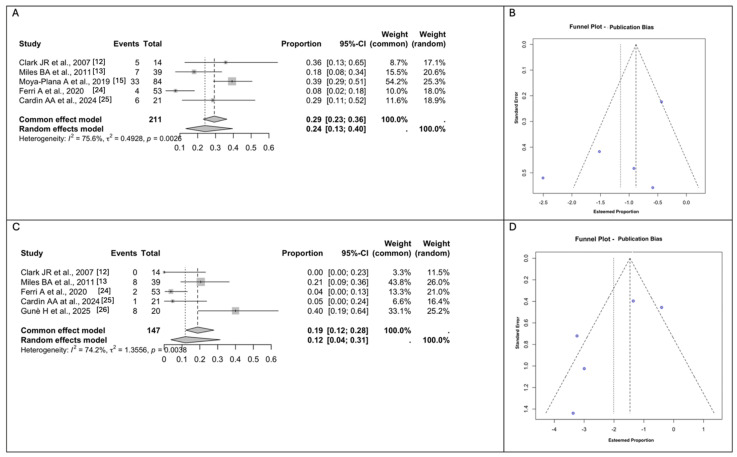
(**A**). Forest plot for Early General Surgical Complications. (**B**). Funnel plots for the evaluation of publication bias using the effect size for General Surgical Complications. (**C**). Forest plot for Fistula Formation. (**D**). Funnel plot for the evaluation of publication bias using the effect size for Fistula Formation. Abbreviations: CI, confidence interval [12,13,15,24,25,26].

Early General Surgical Complications were reported by all the studies included. The Guné et al. [26] study only documented general surgical complications according to the Clavien–Dindo system [28] and, therefore, was excluded from the pooled measurement of Early Surgical Complications. If any complications were classified according to the time of occurrence [13,25], only short-term general surgical complications were considered for the meta-analyses. Considering these assumptions, the rate of pooled Early General Surgical Complications measured using a random effect modeling was 24% (*n* = 17/127; 95% CI 13–40), with a considerable between-study heterogeneity (I^2^ = 75.6% (95% CI: 31.91% to 97.04%), Q =0.4928, *p* = 0.0026). (Figure 2A,B) Total free flap failure occurred in two studies (*n* = 6/231, 2.6%) [15,25]. A total of 57 patients required a revision procedure (*n* = 57/211, 27%) [12,13,15,24,25].

Regarding fistula formation, the rate of pooled Fistula Formation measured using a random effect modeling was 12% (*n* = 19/147; 95% CI 4–31), with a considerable between-study heterogeneity (I^2^ = 74.2% (95% CI: 28.11% to 96.87%), Q = 1.3556, *p* = 0.0038) [12,13,24,25,26]. (Figure 2C,D) The majority were oronasal fistulas (*n* = 10), eight were palatal and one was paranasal. Moya et al. [15] did not provide any information about fistula formation.

### 3.5. Meta-Analyses of Donor Site Morbidity

**Figure 3:**
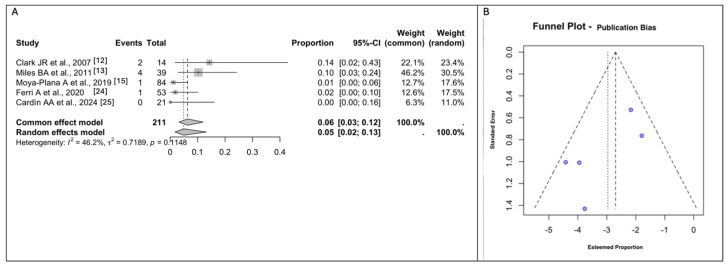
(**A**). Forest plot for Donor Site Morbidity. (**B**). Funnel plots for the evaluation of publication bias using the effect size for Donor Site Morbidity. Abbreviations: CI, confidence interval [12,13,15,24,25].

Five out of the six studies included reported donor site morbidity [12,13,15,24,25]. The rate of pooled Donor Site Morbidity measured using a random effect modeling was 5% (*n* = 8/211; 95% CI 2–13), with a moderate between-study heterogeneity (I^2^ = 46.2% (95% CI: 0.00% to 93.48%), Q = 0.7189, *p* = 0.1148). (Figure 3A,B) The most frequent complication described was seroma (*n* = 6/8; 75%).

Four out of the six studies evaluated the Functional Performance of the Upper Limb after maxillary reconstruction with an SFF using the DASH score [12,13,15,25]. However, Cardìn et al. [25] used the QuickDASH score, whereas Moya et al. [15] did not report the exact DASH score; therefore, these studies were excluded from the meta-analyses. A total of 38 patients filled the DASH score questionnaire, with the mean DASH score being 10.49 (*n* = 38/231; 95% CI 9.47–11.51).

### 3.6. Meta-Analyses of Functional and Aesthetic Outcomes

Unlike the other studies, which assessed function in the entire original sample, Cardìn et al. [25] conducted the evaluation on 16 of the 21 patients who were still alive.

**Figure 4:**
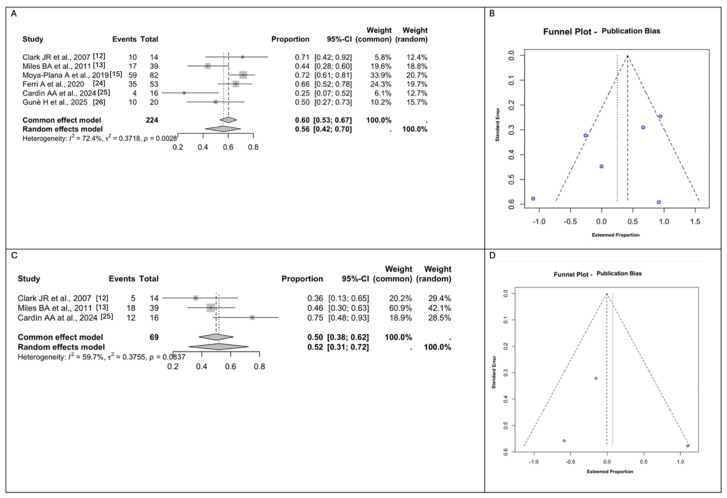
(**A**). Forest plot for Dental Rehabilitation. (**B**). Funnel plot for the evaluation of publication bias using the effect size for Dental Rehabilitation. (**C**). Forest plot for Normal Diet. (**D**). Funnel plot for the evaluation of publication bias using the effect size for Normal Diet. Abbreviations: CI, confidence interval [12,13,15,24,25,26].

All the included studies reported on any Dental Rehabilitation investigated. A total of 35 patients were treated with dental implants (*n* = 35/224, 15.6%) and 100 patients with other types of prosthetic rehabilitation (i.e., dentures, obturators, or nasal prostheses) (*n* = 100/224, 44.6%). The rest of the sample did not receive any dental restoration (*n* = 89/224, 39.7%). The rate of pooled dental rehabilitation measured using random effect modeling was 56% (*n* = 135/224; 95% CI 42–70), with a substantial between-study heterogeneity (I^2^ = 72.4% (95% CI: 29.23% to 95.42%), Q = 0.3718, *p* = 0.00028) (Figure 4A,B).

A total of 3 out of the 5 studies investigated dietary intakes [12,13,25]. A total of 35 patients obtained a Normal Diet after reconstructive surgery (*n* = 35/69, 50.7%), while 34 patients were able to maintain a soft-to-firm diet (34/69, 49.2%%). The rate of a pooled normal diet measured using random effect modeling was 52% (*n* = 35/69; 95% CI 31–72), with a substantial between-study heterogeneity (I^2^ = 59.7% (95% CI: 0.00% to 98.98%), Q = 0.3755, *p* = 0.0837) (Figure 4C,D).

**Figure 5:**
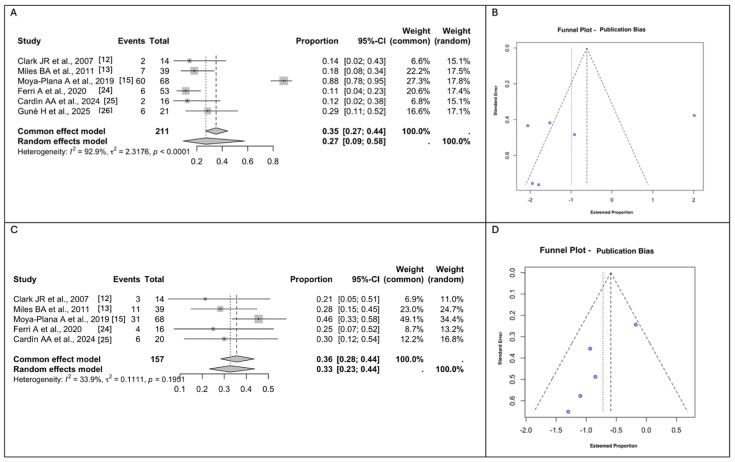
(**A**). Forest plot for Aesthetic Compromise. (**B**). Funnel plot for the evaluation of publication bias using the effect size for Aesthetic Outcomes. (**C**). Forest plot for Eye Problems. (**D**). Funnel plot for the evaluation of publication bias using the effect size for Eye Problems. Abbreviations: CI, confidence interval [12,13,15,24,25,26].

All the studies included reported the aesthetic results, such as an over-contoured or under-contoured midface. Ferri et al. [24] assessed the aesthetic outcomes based on patient self-evaluation, categorizing them as ‘excellent’, ‘good’, ‘poor’, or ‘unacceptable’. Similarly, Moya et al. [15] performed an aesthetic evaluation in 68 patients, grading the results as good, acceptable, or mediocre. For our meta-analyses of the outcome, an aesthetic compromise of ‘excellent’ or ‘good’ was considered as a negative response, while ‘poor’ and ‘unacceptable’ were considered positive. On the other hand, Clark et al. [12] classified the aesthetic outcomes based on the time from surgery, distinguishing between early and late outcomes. For our meta-analysis of the aesthetic compromise, we included only early outcomes, as the late ones were deemed unpredictable by the authors themselves. Given these considerations, the rate of pooled Aesthetic Compromise measured using a random effect modeling was 27% (*n* = 83/211; 95% CI 9–58), with a high between-study heterogeneity (I^2^ = 92.9% (95% CI: 81.79% to 98.82%), Q =2.3176, *p* <0.0001) (Figure 5A,B).

Eye problems were investigated by all the studies included, except for Ferri et al. [12,13,15,25,26]. The rate of pooled Eye Problems measured using random effect modeling was 33% (*n* = 55/157; 95% CI 23–44), with a moderate between-study heterogeneity (I^2^ = 33.9% (95% CI: 0.00% to 92.00%), Q = 0.1111, *p* = 0.1951). Eye problems included dystopia, ectropion, epiphora, enophthalmos, and lagophthalmos (Figure 5C,D).

Only one study assessed patient-reported health-related quality of life outcomes, and, therefore, no pooled estimate was performed [26].

### 3.7. Risk of Bias Assessment

The Newcastle–Ottawa Quality Assessment Scale scores of the individual studies are shown in Table 4. Visual inspection and the Egger’s linear regression test showed a symmetric distribution of the points in the funnel plots for Early General Surgical Complications (t = 1.0374, *p* = 0.2995), Fistula Formation (t = 3.1182, *p* = 0.0525), Donor Site Morbidity (t = 1.0374, *p* = 0.2995), Dental Rehabilitation (t = −1.0352, *p* = 0.3006), Normal Diet (t = 0.0796, *p* = 0.9495), Aesthetic Compromise (t = −1.0927, *p* = 0.2745), and Eye Problems (t = −1.9633, *p* = 0.0496), suggesting no obvious publication bias for Eye Problems.

## 4. Discussion

The midface—encompassing the palate, cheek, maxilla, upper lip, orbit, and nose—serves as both the structural and aesthetic centerpiece of the face. In particular, the maxilla plays a pivotal role, acting as the keystone that bridges the skull base and occlusal plane. It withstands masticatory forces, anchors the dentition, separates the oral and nasal cavities, supports the orbital globe, and provides the foundation for the facial contours and mimetic musculature. The distinct facial identity of each individual is largely influenced by the maxilla’s intricate interplay with the surrounding soft and hard tissues. Consequently, maxillectomy results in significant vital, functional, and aesthetic challenges that must be addressed through a well-planned reconstructive strategy [1,2,3]. Additionally, the management of midface malignancies often necessitates a multimodal approach, with a high incidence of adjuvant treatments such as radiotherapy and chemotherapy [29].

Traditionally, palatal obturators have been the most common method for the rehabilitation of limited maxillectomy defects. This approach offers several benefits, including a reduced operative time, shorter post-operative hospital stays, and an unobstructed visualization of the maxillectomy cavity, which facilitates oncological surveillance [11]. However, obturators also come with significant drawbacks. Patients may experience hypernasality, oronasal leakage, nasal regurgitation of food and liquids, challenges in maintaining hygiene within the maxillectomy cavity, and the need for frequent prosthesis adjustments due to ongoing changes in the size and shape of the palatal defect, particularly in patients undergoing radiation therapy [30,31]. Additionally, larger defects present greater complexity in both functional and aesthetic rehabilitation and are more difficult to manage, as the weight of the prosthesis can hinder retention, especially in partially or fully edentulous patients [27,32].

Given these limitations, various local and pedicled regional flaps have been explored for maxillary reconstruction, with mixed results. For smaller defects, nasal septal flaps, tongue flaps, buccal mucosal flaps, and pharyngeal flaps have been used [33,34,35]. For larger defects, temporalis flaps, forehead flaps, and deltopectoral flaps have been described [36,37]. However, the volume of some of these soft tissue flaps can hinder the retention of dentures, interfere with mastication, and complicate speech articulation.

With the advent of microvascular free tissue transfer, maxillary reconstruction has changed radically, allowing for a precise orientation, shaping, and insertion of the flap to suit the specific defect. This technique also enables the reconstruction to be completed in a single-stage procedure and, when bone is included, facilitates dental restoration using osseo-integrated implants even in irradiated tissues [7,8,38].

Among the various free flaps, the thoracodorsal artery composite flap with a scapular tip stands out for its versatility, offering a substantial amount of bone, multiple independent skin paddles, and a long pedicle (up to 14 cm), which is particularly beneficial for complex maxillofacial reconstructions [12,13,14,24,25,26]. The broader subscapular system of flaps is highly adaptable, allowing for the simultaneous harvest of two skin paddles, two separate bone grafts, and a muscle paddle—all on a single pedicle. This makes it a powerful reconstructive tool, particularly for cases requiring both soft tissue coverage and bony support for future dental rehabilitation [12,13]. Additionally, the subscapular system of flaps offers different composite options, as it allows for multiple tissue components—including muscle, skin, and bone. This system includes various muscle flaps (teres major, latissimus dorsi, serratus anterior, and subscapularis), skin flaps (scapular, parascapular, latissimus dorsi musculocutaneous, and thoracodorsal artery perforator), and bone flaps (lateral scapula, medial scapula, scapular angle, and rib osseous). This flexibility contrasts with flaps like the fibula, iliac crest, and radial forearm, which have more limited composite options and do not allow for an independent rotation of the skin paddle from the bone [5,6,9,13]. As a result, the subscapular system is the preferred option for extensive through-and-through defects that require a three-dimensional management of bone, muscle, and skin, allowing for a wide range of flap movement [24].

A noteworthy innovation in recent years is the Simplified Zygomatic Implant Perforated (ZIP) flap, which offers a streamlined solution for reconstructing low-level maxillary defects, particularly in patients unsuitable for traditional reconstructive methods [39]. Combining soft tissue reconstruction with zygomatic implants allows for early prosthetic loading without the need for complex bone grafting or microvascular surgery. This approach is particularly beneficial in medically compromised patients, offering reduced morbidity and operative time [39]. However, long-term outcomes remain to be fully established.

There are many studies in the literature that have investigated the surgical and functional outcomes of the scapular free flap for the reconstruction of maxillary defects [12,13,15,24,26]. This meta-analysis aims to evaluate the surgical and functional outcomes of using scapular free flaps for maxillary reconstruction.

The pooled data from this meta-analysis reveal a generally low rate of early surgical complications (24%), with a relatively low rate of total free flap failure (six cases across the studies). In the series of Clark et al. [12], one patient developed deep vein thrombosis and pulmonary embolism on the fifth day after surgery (it is worth noting that his patient had travelled on an international flight two days before the procedure.

Moya Plana et al. [15] documented five cases of total necrosis of the flap and three isolated bone necroses. The incidence of total necrosis was higher in patients who had received prior treatment, such as chemoradiotherapy (CT-RT) and/or surgery, highlighting the advantages of primary reconstruction. These findings align with previous studies, which had identified intra-operative fluid use exceeding 7 L, a surgical duration exceeding 10 h, the involvement of multiple microsurgeons, and post-operative radiotherapy as key prognostic factors for free flap failure [40,41]. Additionally, multiple studies have emphasized the importance of the learning curve in this complex surgical procedure, suggesting that a surgeon needs to perform at least 70 procedures to be considered an “expert” [41,42]. However, achieving this threshold is challenging due to the rarity of this malignancy and the high variability in reconstructive defects.

Ferri et al. [24] reported two cases of partial muscular necrosis, which were successfully treated with local debridement under local anesthesia, and one case of intraoral wound dehiscence with a temporary fistula, requiring nasogastric tube feeding for one week.

The Clavien-Dindo system was used by Guné et al. [26,28] to classify general surgical complications. Grade II adverse effects were most common and affected 70% of patients in their series (*n* = 14).

As concerns fistula formation, the fistula group was heterogeneous with both large and small fistulas, which often healed spontaneously. According to Miles et al. [13], the vascularized muscle inherent to the scapular tip flap and inset intraorally seems to provide an excellent means for the spontaneous closure of a post-operative fistula. Moreover, they found no discernible difference regarding fistula formation and the timing of radiation administration and no significant correlation between fistula formation and prior radiotherapy, suggesting that radiotherapy alone may not increase the surgical risk (*p* = 0.13). However, the study lacked sufficient power to assess definitively the impact of radiation.

Regarding secondary surgical procedures, minor revisions were relatively common in the series of Miles et al. [13], with 41% of patients undergoing secondary procedures, primarily to restore the gingivo-buccal sulcus, which was frequently obliterated post-operatively. They proposed the use of prosthetic splints to mitigate this issue. This is in line with Moya Plana et al. [15], who reported that 47.6% of patients required at least one revision surgery. In particular, the most common revision procedures performed in their series were vestibulopathy to facilitate proper dental rehabilitation and surgeries addressing orbito-palpebral disorders.

Long pedicle and chimeric harvesting are often considered the main advantages of the SFF. However, a more important advantage may be the minimal morbidity associated with the use of this flap. In fact, our meta-analysis identified a low pooled donor site morbidity rate. The most frequently observed donor site complication was seroma, which typically did not require any management or need for imaging procedures. This makes the SFF the first option for bone reconstruction in patients who are typically not recommended for other bone-containing free flaps due to factors such as age, comorbidities, vascular anomalies, or extensive composite defects [10].

The assessment of the upper limb functional performance, measured by using the DASH score, showed a mean score of 10.49 (95% CI 12.47–77.82), which is similar to the normative value for the general population, which achieves a mean score of 10.1 [43]. However, not all the patients completed the DASH score questionnaire because they were lost during the follow-up [12,13].

In terms of dental rehabilitation, 56% (*n* = 135/224; 95% CI 42–70) of patients achieved prosthetic restoration, emphasizing the SFF’s ability to provide a stable base for implants. However, a significant portion of patients did not undergo prosthetic rehabilitation, often due to factors such as advanced disease, financial constraints, or treatment fatigue [13]. Despite this, 52% (*n* = 35/69; 95% CI 31–72) of patients were able to resume a normal diet [12,13,25].

A high rate of between-study heterogeneity was observed for the item ‘Aesthetic Compromise’. The highest rate of aesthetic compromise was reported in the series of Moya Plana et al. [15], where the majority of the patients (*n* = 49/84, 55.9%) had orbital floor involvement and therefore required orbital floor reconstruction, often leading to both oculopalpebral and aesthetic issues. Overall, the aesthetic results were favorable, with only 27% (*n* = 83/211; 95% CI 9–58) of patients experiencing a significant aesthetic compromise, reflecting the flap’s adaptability to restore facial contours and symmetry.

Similarly, eye-related problems are correlated with large defects. Reconstructing the orbital floor remains a major surgical challenge since achieving both functional and aesthetic success is particularly difficult, especially after radiotherapy [44,45]. According to Connolly et al. [46], diplopia is related to Brown III defects, and ectropion and epiphora are possibly associated with the Weber–Fergusson incision. To improve outcomes and prevent enophthalmos, Miles et al. [13] suggested using a greenstick fracture technique and plating a section of the thin medial scapular bone to provide additional orbital support. This approach may enhance structural stability and help maintain a proper globe positioning in complex midface reconstructions. Cardìn et al. [21] now incorporate primary lacrimal cannulation and dacryocystorhinostomy into their standardized approach for type III maxillary defects, in order to prevent post-operative epiphora and long-term lacrimal drainage issues.

Only one study [22] assessed the health-related quality of life (HR-QoL) outcomes following maxillary reconstruction with the scapular osseous free flap. Guné et al. reported patient-reported outcome measures (PROMs) from 20 maxillary reconstructions with the scapular osteomyocutaneous free flap, using the FACE-Q questionnaire [47] and focusing on three specific surgical outcomes: dental rehabilitation, oronasal fistula, and eye problems. A key strength of this study is the 100% survey response rate. Additionally, the questionnaires were completed 12 to 60 months post-operatively, providing a sufficient timeframe for the assessment of long-term HR-QoL. In this study, patients who had undergone dental rehabilitation showed a trend toward improved facial function scores and a higher HR-QoL related to speaking distress. Patients who had developed post-operative fistulas showed a trend towards worse facial function in eating, drinking, and oral competence. However, no significant differences in speaking function or HR-QoL were observed between the fistula and non-fistula groups.

According to Moya Plana et al. [15], soft palate surgery is associated with a poor prognosis for speech (*p* < 10^−4^) and swallowing (*p* = 0.005) disorders. In contrast, these functional impairments were absent when the soft palate was preserved. In such cases, autologous fat transfer offered a partial improvement, though multiple procedures were often required.

### 4.1. Limitations

This meta-analysis has several limitations that must be acknowledged. First, there was considerable heterogeneity among the included studies, particularly in outcomes related to aesthetic compromise and fistula formation. This variability may be attributed to differences in study designs, patient populations, and defect characteristics. Additionally, small sample sizes in some studies limit the statistical power and generalizability of the findings. Selection bias is another concern, as many studies were retrospective and non-randomized, potentially influencing the outcomes reported. Moreover, many of the included studies originated from high-volume centers with specific expertise in scapular flap reconstruction. This introduces a potential reporting bias, as favorable outcomes may reflect institutional proficiency rather than the intrinsic advantages of the flap itself. This variability in surgical experience across centers further complicates the interpretation of results.

The selection process itself also presents limitations. Although the inclusion criteria were broad, many studies were excluded because outcome data for SFFs could not be clearly isolated, or when the studies did not meet essential methodological quality standards. This reduced the number of eligible studies, leading to a limited sample size for the meta-analysis. While this exclusion process aimed to improve the quality of the included studies, it may also have resulted in the omission of potentially relevant data, thus limiting the comprehensiveness of the findings.

The analysis also faced challenges in standardizing outcomes, as various methods were used to assess functional and aesthetic recovery across different studies. Functional and aesthetic outcomes were evaluated using diverse methodologies, with inconsistent reporting of parameters such as donor site morbidity, facial appearance, and symmetry. Similarly, adjuvant treatments—including radiotherapy, chemotherapy, and prior surgeries—were not consistently controlled for, potentially affecting healing and complication rates.

Moreover, many of the included studies had short-term follow-ups, and, therefore, long-term functional and aesthetic outcomes may not have been fully captured. This is particularly important for assessing the durability of the reconstructive results and the potential need for revision surgeries or prosthetic interventions over time. Finally, while the SFF demonstrated significant advantages in terms of reconstructive outcomes, donor site morbidity and potential complications from harvesting the flap were not always fully reported or assessed comprehensively.

### 4.2. Future Directions

To address these limitations, further studies are warranted to assess the long-term outcomes of scapula osseocutaneous flaps in comparison to other reconstructive options, particularly in relation to defect size and patient-centered outcomes such as speech, mastication, and aesthetics. There is also a need for the development of standardized algorithms for flap selection, incorporating defect classification and rehabilitation strategies. Emerging techniques such as the simplified ZIP flap offer promising alternatives and should be evaluated through prospective studies to determine their efficacy and limitations in maxillary defect reconstruction.

## 5. Conclusions

This systematic review and meta-analysis confirm that the SFF is a reliable option for maxillary reconstruction, offering low donor site morbidity and low rates of fistula formation. It provides effective functional restoration, supports dental rehabilitation, and enables many patients to resume a normal dietary intake. Aesthetic outcomes are generally favorable, particularly in cases involving complex composite defects where both bone and soft tissue reconstruction are required. The SFF is especially suitable for patients with comorbidities or anatomical considerations that preclude the use of other osseous free flaps. Overall, the SFF represents a versatile and dependable reconstructive option with a favorable complication profile.

## Figures and Tables

**Table 1:** Search strategy for each database. Abbreviations: SFF = scapular free flap; RCT = randomized clinical trials.

Database	Search Terms	Filters Applied	Last Search Date
PubMed/MEDLINE	(“scapular free flap” OR “SFF” OR “scapular flap” OR “free flap” AND (“maxillary reconstruction” OR “maxillectomy” OR “maxillary defect”) AND (“surgical outcome” OR “functional outcome” OR “aesthetic outcome” OR “complication” OR “fistula” OR “morbidity” OR “dental rehabilitation” OR “upper limb function” OR “eye problem” OR “revision” OR “flap survival”) AND (“cohort study” OR “case–control study” OR “cross-sectional study” OR “randomized clinical trial” OR “RCT”)	Full text, English language, Published in a peer-reviewed journal	10th February 2025
Cochrane Library	(“scapular free flap” OR “SFF” OR “scapular flap”) AND (“maxillary reconstruction” OR “maxillectomy”) AND (“surgical outcome” OR “functional outcome” OR “aesthetic outcome” OR “complication” OR “fistula” OR “morbidity” OR “dental rehabilitation” OR “upper limb function”) AND (“cohort” OR “case–control” OR “RCT”)	Peer-reviewed articles, English language	8th February 2025
Google Scholar	“scapular free flap” AND “maxillary reconstruction” AND (“surgical outcome” OR “functional outcome” OR “aesthetic outcome” OR “fistula” OR “morbidity” OR “dental rehabilitation” OR “upper limb function”) AND (“cohort study” OR “case–control” OR “randomized trial” OR “RCT”)	Peer-reviewed articles, English language	10th February 2025
Scopus	(“scapular free flap” OR “SFF” OR “scapular flap”) AND (“maxillary reconstruction” OR “maxillectomy”) AND (“surgical outcome” OR “functional outcome” OR “aesthetic outcome” OR “complication” OR “fistula” OR “morbidity” OR “dental rehabilitation” OR “upper limb function” OR “flap survival”) AND (“cohort” OR “case–control” OR “cross-sectional” OR “RCT” OR “clinical trial”)	Full text, English language, Published in a peer-reviewed journal	10th February 2025

**Table 2:** Eligibility criteria. Abbreviations: SFF = scapular free flap; DASH score = Disabilities of the Arm, Shoulder, and Hand score; RCTs = randomized clinical trials.

Inclusion Criteria	
Patients (P)	Studies including adult patients (≥18 years) who underwent SFF reconstruction following a post-ablative maxillectomy for any cause (e.g., cancer, trauma).
Intervention (I)	Studies where the primary intervention was SFF reconstruction following maxillectomy, including both total and partial maxillectomy procedures.
Outcomes (O)	Studies must have reported on at least one of the following outcomes: Surgical outcomes: such as complications, fistula formation, flap survival, and donor site morbidity. Functional outcomes: including upper limb function (measured with scales such as DASH score), dental rehabilitation, and the ability to resume normal activities (e.g., eating, speaking). Aesthetic outcomes: focusing on the cosmetic results and facial symmetry. Eye-related issues.
Study design (S)	Retrospective and prospective cohort studies, case–control and cross-sectional studies and RCTs.
**Exclusion Criteria**	
Non-Full Text availability	To ensure access to complete methodology, data, and results.
Studies that included fewer than 5 patients	To minimize the risk of bias and ensure the robustness of the analysis.
Non-original research	Review articles, case reports, conference abstracts, letters to the editor, and book chapters.

**Table 3:** Summary of included studies. Abbreviations: STFF = scapular tip free flap; LOS = length of hospital stay; DASH score = Disabilities of the Arm, Shoulder, and Hand score.

First Author, Year	Country	Study Design	No. (Male)	Type of Scapular Flap	Classification of Maxillectomy Defects	Median Follow-Up	Objective Outcomes	PROMS Measures
Clark JR et al., 2007 [12]	Canada	Retrospective	14 (8)	Scapular angle osteomyogenous flap	Okay	12	Reconstructive Outcomes: approach, Muscle/other combined with scapular tip, bone orientation/other, LOS day, dental restoration, morbidity, revision, diet	Upper limb function: DASH score
Miles BA et al., 2011 [13]	Canada	Retrospective	39 (N/A)	Scapular angle osteomyogenous flap	Okay	12.5	Reconstructive Outcomes: early and late postoperative complications, dental restoration, revision, diet	Upper limb function: DASH score
Moya-Plana A et al., 2019 [15]	France	Retrospective	84 (46)	Latissimus dorsi-scapular free	Kolb	45	Post- operative complications, and oncologic and functional outcomes (speech and swallowing disorders, dental rehabilitation, rhinologic dysfunction, oculopalpebral disorders, and esthetic outcomes: good, acceptable, or mediocre)	Upper limb function: DASH score
Ferri A et al., 2020 [24]	Italy	Retrospective	53 (29)	STFF: scapular tip free flap	Brown I: 16; II: 21; IIIa: 13; IIIb: 3	N/A	Complications. Functional results: oronasal fistula, achievement of dental rehabilitation, and the extent of mouth opening (good (>3 cm), partially limited (2–3 cm), or limited (<2 cm))	Esthetic outcomes: excellent, good, poor, or unacceptable
Cardìn AA at al., 2024 [25]	Portugal	Retrospective	21 (7)	STFF: scapular tip free flap	Brown IIa: 5; IIb: 5; IIIa: 8; IIIb: 3	41	Surgical outcomes: days of admission, short- and long-term complications, follow-up months, and disease	Functional assessment: speech, eating or swallowing, rhinologic dysfunction, eye or eyelid disorders, orofacial pain, breathing, and esthetic or social discomfort). Upper limb function: QuickDASH score
Gunè H et al., 2025 [26]	Sweden	Prospective cohort	20 (16)	SOFF: osseous flap, osseous flap with ± latissimus dorsi muscle ± skin island flap	Brown I: 1; II: 12; III: 6; IV: 1	6	Postoperative outcomes and complications: general surgical complications according to Clavien–Dindo, dental rehabilitation, oronasal fistula and eye-related problems	Functional, psycho- social, and experiential outcomes: FACE-Q

**Table 4:** Newcastle–Ottawa Quality Assessment Scale scores of the individual studies.

Study	Selection	Comparison	Outcome
Clark JR et al., 2007 [12]	xxx	x	xxx
Miles BA et al., 2011 [13]	xxx	x	xxx
Moya-Plana A et al., 2019 [15]	xxx	x	xxx
Ferri A et al., 2020 [24]	xxx	x	xx
Cardìn AA et al., 2024 [25]	xxx	x	xxx
Gunè H et al., 2025 [26]	xxx	x	xx

## Data Availability

The data that support the findings of this study are available from the corresponding author upon reasonable request.
